# EUPD: A Case Study Highlighting the High Stakes of First Impressions and the Dangers of Groupthink

**DOI:** 10.1192/bjo.2025.10723

**Published:** 2025-06-20

**Authors:** Aisha Duale

**Affiliations:** St Helens and Knowsley NHS Trust, Liverpool, United Kingdom

## Abstract

**Aims:** Emotionally unstable personality disorder (EUPD) is a mental disorder that can be one of the most misunderstood diagnoses. It is a controversial and stigmatised condition among healthcare professionals which may lead to sub-standard levels of care and sub-therapeutic patient experience.

**Methods:** A 47-year-old female was admitted to an inpatient unit four times over five years. She exhibited visual and auditory hallucinations and fixed delusions. This patient had a diagnosis of Paranoid Schizophrenia.

During the fourth admission, she was admitted under Section 2 after she was found walking on the M57 in the middle of the night with suicidal ideation.

The clerking doctor had made note, upon admission, of self-harm behaviours that had occurred five years prior. On the ward, the patient would walk around half-naked and behave bizarrely towards staff and other service users. After discussion, the nursing staff concluded that the patient “knew what she was doing” and must have a diagnosis of EUPD considering her record of self-harm and odd behaviour on the ward. This was then included in the handover sheet, with a query, which was shared amongst ward staff.

The resulting stigma led to staff behaving in a discriminatory manner including avoidance of the patient and unchallenged refusals during medication administration. The negative approach of the ward staff dissipated when the patient’s behaviours settled with an antipsychotic. An informal meeting was conducted with the ward staff to highlight the potential iatrogenic harm that stigma can cause. Staff were evidently remorseful and understood the importance of engaging with all patients with the same standard of care, regardless of diagnosis.

**Results:** The stigma associated with EUPD is so potent that it may filter through to patients without the diagnosis based on loose associations. Healthcare professionals may distance themselves from patients with EUPD which may perpetuate sensitivities such as rejection and abandonment. This study highlights that this stigma poses a risk to the wider patient cohort should similar risk profiles or symptoms be displayed.

**Conclusion:** Mitigating the negative impact of an EUPD diagnosis must start with an acceptance and recognition of the dangers that staff narrative can have on patient care. This case study demonstrates the dangerous impact of stigma whereby a place of safety, the hospital, becomes the antithesis of therapeutic intervention. Of course, a more conclusive outcome would be to revisit the use of this diagnostic label and review policies in the management of EUPD in the inpatient setting.

